# Increased placental expression of cannabinoid receptor 1 in preeclampsia: an observational study

**DOI:** 10.1186/s12884-014-0395-x

**Published:** 2014-12-02

**Authors:** Gergely Fügedi, Miklós Molnár, János Rigó, Júlia Schönléber, Ilona Kovalszky, Attila Molvarec

**Affiliations:** First Department of Obstetrics and Gynecology, Semmelweis University, Baross utca 27, Budapest, H-1088 Hungary; Institute of Pathophysiology, Semmelweis University, Budapest, Hungary; First Department of Pathology and Experimental Cancer Research, Semmelweis University, Budapest, Hungary

**Keywords:** Anandamide, Endocannabinoid, Placenta, Preeclampsia, Pregnancy

## Abstract

**Background:**

The endocannabinoid system plays a key role in female reproduction, including implantation, decidualization and placentation. In the present study, we aimed to analyze cannabinoid receptor 1 (CB1), CB2 and fatty acid amid hydrolase (FAAH) expressions and localization in normal and preeclamptic placenta, in order to determine whether placental endocannabinoid expression pattern differs between normal pregnancy and preeclampsia.

**Methods:**

Eighteen preeclamptic patients and 18 normotensive, healthy pregnant women with uncomplicated pregnancies were involved in our case–control study. We determined CB1, CB2 and FAAH expressions by Western blotting and immunohistochemistry in placental samples collected directly after Cesarean section.

**Results:**

CB1 expression semi-quantified by Western blotting was significantly higher in preeclamptic placenta, and these findings were confirmed by immunohistochemistry. CB1 immunoreactivity was markedly stronger in syncytiotrophoblasts, the mesenchymal core, decidua, villous capillary endothelial and smooth muscle cells, as well as in the amnion in preeclamptic samples compared to normal pregnancies. However, we did not find significant differences between preeclamptic and normal placenta in terms of CB2 and FAAH expressions and immunoreactivity.

**Conclusions:**

We observed markedly higher expression of CB1 protein in preeclamptic placental tissue. Increased CB1 expression might cause abnormal decidualization and impair trophoblast invasion, thus being involved in the pathogenesis of preeclampsia. Nevertheless, we did not find significant differences between preeclamptic and normal placental tissue regarding CB2 and FAAH expressions. While the detailed pathogenesis of preeclampsia is still unclear, the endocannabinoid system could play a role in the development of the disease.

## Background

Preeclampsia, characterized by hypertension and proteinuria developing after the 20^th^ week of gestation in a previously normotensive woman, is a severe complication of human pregnancy with a worldwide incidence of 3-8% [1]. It is among the leading causes of maternal, as well as perinatal morbidity and mortality, even in developed countries. Despite extensive research, the etiology and pathogenesis of preeclampsia are not completely understood. To our current knowledge, preeclampsia is rather a syndrome than a single disease, originating either from poorly developed placenta (placental preeclampsia) or a maternal constitution described by abnormal systemic inflammatory response based on microvascular dysfunction (maternal preeclampsia) [2,3]. The two-stage model of placental preeclampsia consists from abnormal placental development (stage 1) due to insufficient trophoblast invasion, where invasive extravillous cytotrophoblasts fail to remodel the maternal spiral arteries in the placenta, causing hypoxia and oxidative stress through impaired uteroplacental circulation. Poor placentation between 6 to 18 weeks of gestation is followed by systemic maternal symptoms, such as hypertension, proteinuria, clotting and liver dysfunction caused by the increasingly hypoxic placenta (stage 2) [4].

Endocannabinoids (ECs) are endogenous ligands binding to the similar receptor as delta-9-tetrahidrocannabinol (Δ^9^-THC), the most potent psychotropic constituent of marijuana. The G protein-coupled cannabinoid receptors (CB1 and CB2) exert their effects through various signaling pathways including adenylyl cyclase inhibiton leading to diminished cyclic adenosine monophosphate (cAMP) levels, activation of mitogen-activated protein kinases (MAPK), and either activation or inhibition of various ionic channels [5]. The most studied endogenous CB-receptor ligand anandamide (AEA) is synthesized on demand from the lipid precursor *N-*arachidonoyl phosphatidylethanolamine (NAPE) by a specific phospholipase D enzyme (NAPE-PLD), and is primarily degraded by fatty acid amid hydrolase (FAAH) to ethanolamine and arachidonic acid. ECs are not stored, thus hydrolyzing enzymes play important role regulating extracellular ligand levels [6].

AEA and CB1 are localized mainly in the central nervous system, but also detected in the adrenal gland, heart, uterus, testis and liver, whereas CB2 is primarily restricted to immune and hematopoetic cells, and the spleen [7]. This wide variety of localization implicates that the endocannabinoid system (ECS) is responsible for several pathophysiological processes: modulation of ECS affects almost all human diseases such as obesity; diabetes and diabetic complications; neurodegenerative, inflammatory, cardiovascular, liver, gastrointestinal and skin diseases; pain and psychiatric disorders; cachexia and cancer amongst many others [8]. Regarding the female reproductive system, accumulating evidence shows that CB-receptors, FAAH and ECs present in the ovary, oviduct, uterus, embryo and placenta play a key role in female reproduction, including oogenesis, preimplantation embryo development, oviductal embryo transport, implantation, decidualization and placentation, pregnancy maintenance and labor [5,9,10]. A recent study has shown that tightly regulated EC levels are critical for female reproductive success, as both silenced and elevated AEA levels result in unidirectional gene expression changes, causing impaired trophoblast migration ability [11]. Use of marijuana during gestation results in fetal growth restriction, also suggesting the significance of the ECS in pregnancy [12,13].

Given the role of the endocannabinoid system in implantation, decidualization and placentation, in the present study we aimed to analyze CB1, CB2 and FAAH expressions and localization in normal and preeclamptic placenta, in order to determine whether placental endocannabinoid expression pattern differs between normal pregnancy and preeclampsia.

## Methods

### Study participants

Eighteen preeclamptic patients and 18 normotensive, healthy pregnant women with uncomplicated pregnancies were involved in our case–control study. The study participants were enrolled in the First Department of Obstetrics and Gynecology at the Semmelweis University, Budapest, Hungary. All women were Caucasian and resided in the same geographic area in Hungary. Exclusion criteria were multifetal gestation, chronic hypertension, diabetes mellitus, autoimmune disease, angiopathy, renal disorder, maternal or fetal infection and fetal congenital anomaly.

Preeclampsia was defined by increased blood pressure (≥140 mmHg systolic or ≥90 mmHg diastolic on ≥2 occasions at least 6 hours apart) that occurred after the 20^th^ week of gestation in a woman with previously normal blood pressure, accompanied by proteinuria (≥0.3 g/24 h or ≥1 + on dipstick in the absence of urinary tract infection). Blood pressure returned to normal by the 12^th^ postpartum week in each preeclamptic study patient. Preeclampsia was regarded as severe if any of the following criteria was present: blood pressure ≥160 mmHg systolic or ≥110 mmHg diastolic, or proteinuria ≥5 g/24 h (or ≥3 + on dipstick). Early onset of preeclampsia was defined as onset of the disease before the 34^th^ week of gestation (between the 20^th^ and 33^rd^ completed gestational weeks) [14]. Fetal growth restriction was diagnosed if the fetal birth weight was below the 10^th^ percentile for gestational age and gender, based on Hungarian birth weight percentiles.

The study protocol was approved by the Regional and Institutional Committee of Science and Research Ethics of the Semmelweis University, and written informed consent was obtained from each patient. The study was conducted in accordance with the Declaration of Helsinki.

### Placental samples

Placental tissue samples were collected directly after Cesarean section from a single site between the placental rim and cord insertion, free of visible infarction and calcification. The median gestational age was 38 weeks in the control and 37 weeks in the preeclamptic group (Table 1). One piece of the placental tissue sample was placed into a plain tube without fixation, and stored at −80°C until the measurements. A second piece of the sampled placental tissue was fixed in 10% neutral-buffered formalin for 4 days before embedding in paraffin wax.

**Table 1:** **Clinical characteristics of healthy pregnant women and preeclamptic patients**

	**Healthy pregnant women (n = 18)**	**Preeclamptic patients (n = 18)**	**Statistical significance (p value)**
Age (years)	32 (28–35)	29 (28–32)	NS
Pre-pregnancy BMI (kg/m^2^)	21.8 (18.7-26.4)	24.2 (21.2-29.6)	NS
Smokers	1 (5.6%)	1 (5.6%)	NS
Primiparas	6 (33.3%)	13 (72.2%)	<0.05
Systolic blood pressure (mmHg)	120 (110–120)	180 (166–180)	<0.001
Diastolic blood pressure (mmHg)	80 (70–80)	110 (100–110)	<0.001
Gestational age at delivery (weeks)	38 (34–39)	37 (35–38)	NS
Fetal birth weight (grams)	3100 (2300–3370)	2475 (1910–3400)	NS
Fetal growth restriction	0 (0%)	4 (22.2%)	NS

Data are presented as median (interquartile range) for continuous variables and as number (percentage) for categorical variables.

*NS*: Not significant; *BMI*: Body mass index.

### Western blot

The Western blot was performed on full-thickness blocks from the chorionic plate to the basal plate in order to determine the overall expression of CB1, CB2 and FAAH in the placenta. One g of placental tissue was minced well and homogenized in 10 ml of lysis buffer (20 mM Tris–HCl, 5 mM EDTA, 10 mM EGTA, 2 mM DTT, 1 mM Na_3_VO_4_, 25 μg/ml PMSF, 2.5 μg/ml Leupeptin, 2.5 μg/ml Aprotinin, 625 μM sodium-pyrophosphate, 1 mM β-glycerophosphate, 0,1% Triton) by a homogenizer for 3 × 15 sec. The homogenate was centrifuged at 800 *g* for 15 min at 4°C, and the pellet was discarded. The supernatant was collected and stored at −80°C and used within four weeks for assays. To assess FAAH (AT1983a, mouse monoclonal antibody, Abgent Inc., San Diego, CA, USA), CB1 (EB06945 goat polyclonal anti-CB1 antibody, Everest Biotech, England) and CB2 (EB06946 goat polyclonal anti-CB2 antibody, Everest Biotech, England) expressions in human placenta, 60 μg of extracted proteins were used for Western blot analysis.

Samples were prepared in 2x Laemmli buffer containing 100 mM dithiothreitol and boiled in a water bath for 15 min. Protein (60 μg) was separated on a SDS-PAGE (9 %) gel followed by a wet transfer to a nitrocellulose membrane for 90 min. We used Ponceau S to determine whether proteins migrated uniformly onto the nitrocellulose membrane. After gently rinsing, the membranes were blocked for 1 h at room temperature in 10% (wt/vol) non-fat dried milk in Tris-buffered saline (TBS) with 0.1% Tween 20 (TBST) and then incubated overnight with antibody against the CB1 or CB2 or FAAH, respectively. The antibodies were diluted (CB1 1:1000, CB2 and FAAH 1:500) in 1% bovine serum albumin in TBST. Blots were incubated in a HRP-conjugated secondary antibody in TBST for 1 h at room temperature and visualized by ECL-Western blotting detection system (Amersham Pharmacia Biotech, England). Mouse testicular homogenate and color molecular weight markers were run parallel with the samples and were used to identify the specific bands. The membrane was stripped at 60°C for 30 min in stripping buffer (100 mM 2-mercaptoetanol, 2% SDS and 62.5 mM Tris–HCl, pH 7.6), and were reprobed with MAPK1 antibody (1:1000) to normalize for loading.

Western blot signals were semi-quantified by densitometry analysis using a GELDOC 1.00-UV system (Biorad, Hercules, CA, USA). The signals of the specific bands in densitometry unit were adjusted according to the changes of the corresponding density of MAPK1 bands on the same loaded membrane. The deviation of the density of MAPK1 bands from the mean was used to normalize the value of the CB1, CB2 or FAAH bands. The corrected signals of the preeclamptic placentas were expressed as % of the mean values of normal placentas.

### Immunohistochemistry (IHC)

Immunohistochemical staining was performed on 16 placental samples from each study group. Anti-CB1 (GTX100219) and anti-CB2 (GTX101357) rabbit polyclonal antibodies were obtained from GeneTex (Irvine, CA, USA), whereas anti-FAAH (AT1983a) mouse monoclonal antibody was purchased from Abgent (San Diego, CA, USA). In comparison to Western blot, different CB-antibodies were chosen due to the incompatibility of the antibodies used during Western blot with IHC technology. Tissue sections (3 μm thick) were mounted onto SuperFrost Ultra Plus Adhesion Slides (Thermo Scientific, USA), dried in thermostat at 56°C for 1 h, then 24 h at room temperature before use. Immunostaining process was carried out with Leica BOND-MAX fully automated IHC & ISH system (Leica Biosystems, St. Louis, MO, USA), using Bond Polimer Refine Detection kit (Leica Biosystems), which included peroxide block (3% Hydrogen peroxide), post primary polymer penetration enhancer (10% animal serum in Tris-buffer saline and 0.09% ProClin™ 950), polymer Poly-HRP anti-mouse/rabbit IgG (each at 8 μg/ml, containing 10% animal serum in Tris-buffer saline and 0.09% ProClin™ 950), DAB Part 1 (66 mM 3,3’-Diaminobenzidine tetrahydrochloride, in stabilizer solution), DAB Part B (0.05% Hydrogen Peroxide in a stabilizer solution) and Hematoxylin (0.02%). Slides were dewaxed three times with Bond Dewax Solution (Leica Biosystems) at 72°C, then rehydrated in three steps with graded alcohol and washed with buffer solution (Bond Wash Solution, Leica Biosystems). Antigen retrieval for CB1, CB2 and FAAH was performed by incubating slides with Leica Bond Epitope Retrieval Solution 2 (pH 9.0) for 20 min. Primary antibodies diluted in Bond Primary Antibody Diluent (Leica Biosystems) to 1:1000 for CB1 and CB2, and 1:1200 for FAAH were added for 20 min, then slides were incubated with post primary polimer for 15 min. After washing slides with buffer solution and deionized water, peroxidase activity was blocked by incubation with peroxide block for 3 min. After additional washing (buffer and deionized water), mixed DAB refine was added to slides for 10 min, followed by deionized washing for three times, and incubation with Hematoxylin for 4 min. In between steps, sections were washed with Leica Wash Solution 10x Concentrate (diluted to 1:9 ratio). We used human cerebellum as a positive control.

Images were taken on a Zeiss Axiolmager A2 microscope equipped with an AxioCam ICc 1 camera (Carl Zeiss Ltd., NY, USA) connected to a computer running AxioVision image capture and processing software (version release 4.8.2., Carl Zeiss Ltd.), captured at 200x and 400x magnification.

### Statistical analysis

The normality of continuous variables was assessed using the Shapiro-Wilk’s *W*-test. As the continuous variables were not normally distributed, non-parametric statistical methods were applied. To compare continuous variables between two groups, the Mann–Whitney *U*-test was used. The Fisher exact and Pearson χ^2^ tests were performed to compare categorical variables between groups.

Statistical analyses were carried out using the following software: STATISTICA (version 11; StatSoft, Inc., Tulsa, Oklahoma, USA) and Statistical Package for the Social Sciences (version 22 for Windows; SPSS, Inc., Chicago, Illinois, USA). For all statistical analyses, p < 0.05 was considered statistically significant.

In the article, data are reported as median (interquartile range) for continuous variables and as number (percentage) for categorical variables.

## Results

### Patient characteristics

The clinical characteristics of the study participants are presented in Table 1. There were no statistically significant differences in terms of maternal age, pre-pregnancy body mass index (BMI), gestational age at delivery and fetal birth weight and the percentage of smokers between the two study groups. The systolic and diastolic blood pressures, as well as the percentage of primiparas, were significantly higher in the preeclamptic group than in the control group. Fetal growth restriction was absent in healthy pregnant women, whereas it occurred in 4 cases in the preeclamptic group. Fourteen women had severe preeclampsia and 5 patients experienced early onset of the disease.

### Western blot

**Figure 1:**
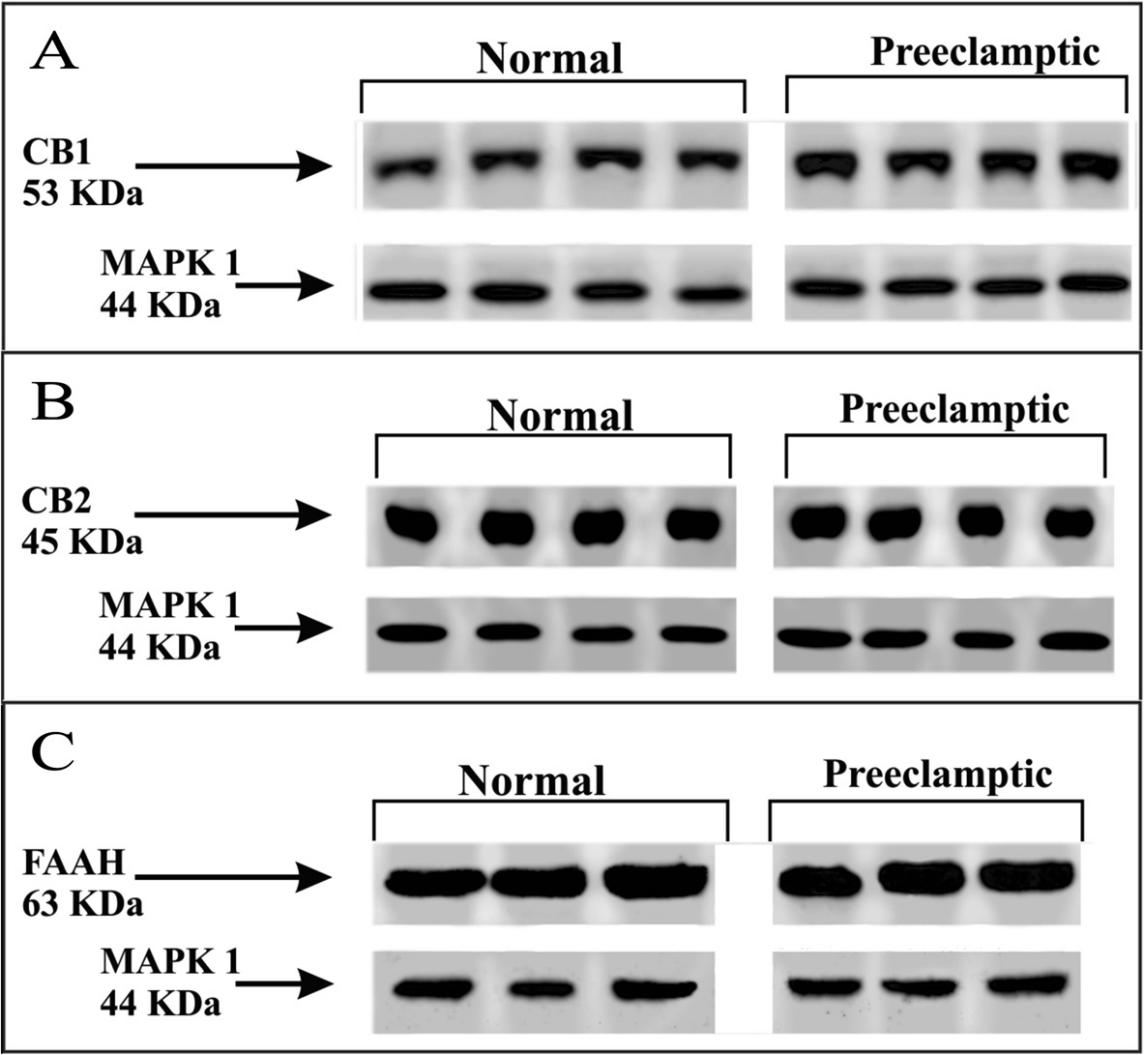
**Representative Western blotting demonstrating CB1 (A), CB2 (B) and FAAH (C) expressions in normal and preeclamptic placental tissue.**

**Figure 2:**
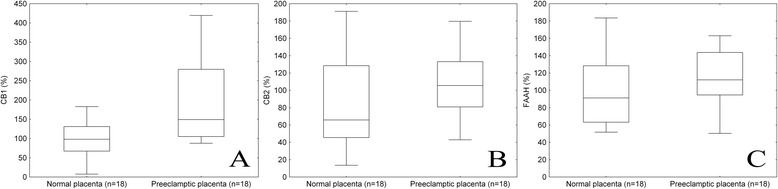
**Densitometry analysis of CB1 (p = 0.008, A), CB2 (p > 0.05, B) and FAAH (p > 0.05, C) expressions in normal and preeclamptic placental tissue.** Middle line: median; Box: interquartile range (25–75 percentile); Whisker: range (excluding outliers).

Representative Western blotting of CB1, CB2 and FAAH expressions in normal and preeclamptic placental tissue is shown in Figure 1. According to densitometry analysis, placental expression of CB1 protein was significantly higher in preeclamptic patients than in normotensive, healthy pregnant women (149.3 (105.0-279.7) % versus 98.1 (67.3-131.0) %, p = 0.008; Figure 2A). Nevertheless, no significant differences were observed in placental CB2 (105.5 (80.9-133.2) % versus 65.8 (45.5-128.4) %, p > 0.05; Figure 2B) and FAAH (112.2 (94.7-143.8) % versus 91.2 (63.3-128.4) %, p > 0.05; Figure 2C) protein expressions between the two study groups.

### Immunohistochemistry

#### CB1 immunoreactive staining patterns

**Figure 3:**
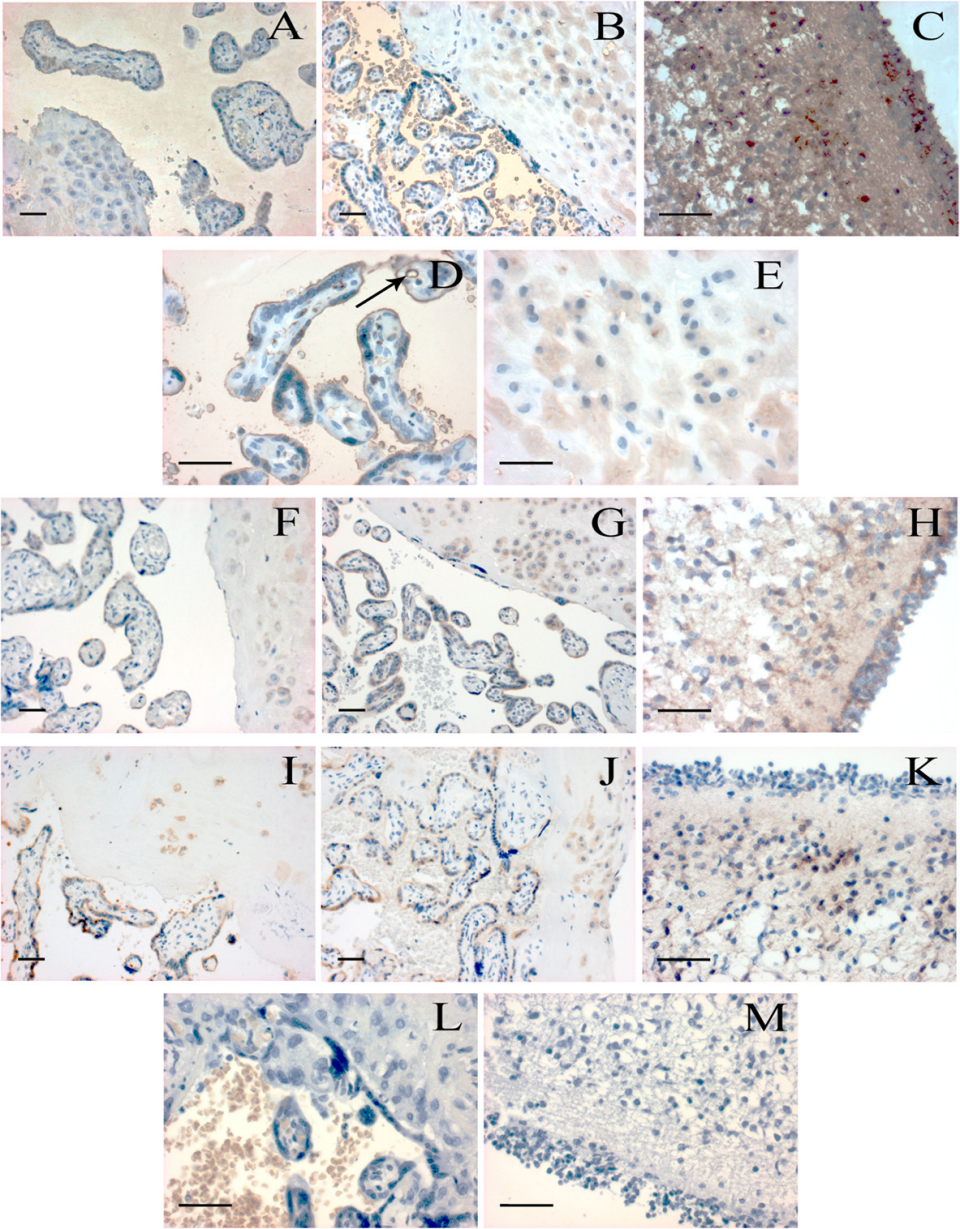
**Localization of CB1, CB2 and FAAH in human placental villi, decidua and cerebellum.** CB1 specific staining is shown in normal **(A)** and preeclamptic **(B)** placental tissues, images were captured at 200× magnification. Further images demonstrate strong CB1 positivity in preeclamptic placental villi **(D)** and decidua **(E)** at 400× magnification. Arrow indicates strong CB1 immunoreactivity in villous capillary endothelial cells. Positive control reaction for CB1 is revealed in human cerebellum **(C)**. CB2 specific staining is shown in normal **(F)** and preeclamptic **(G)** placental tissues, and in human cerebellum **(H)** as a positive control. FAAH specific staining is presented in normal **(I)** and preeclamptic **(J)** placental tissues, and in human cerebellum **(K)** as a positive control. Negative controls are shown in human placental villi and decidua **(L)**, and in human cerebellum **(M)**. Placental tissue samples from normal pregnancy and preeclampsia were captured at 200× magnification, while positive and negative controls were taken at 400× magnification; bar =100 μm.

Strong CB1 immunoreactivity was detected in the syncytiotrophoblast layer, as well as in the endothelial cells of blood vessels. Less intense, but positive staining was found in the decidua, capillary smooth muscle cells, stromal fibroblasts and in the amnion. CB1 immunoreactivity in the previously mentioned localizations was observed both in normal and preeclamptic placenta, however, staining was markedly more intense in preeclampsia (Figure 3A-E).

#### CB2 immunoreactive staining patterns

Immunoreactive CB2 was detected both in normal and preeclamptic placenta, but there was no difference in the staining intensity and localization between the two groups. Syncytiotrophoblasts and stromal fibroblasts showed positive reaction for CB2, although staining was less strong compared to CB1. CB2 immunoreactivity was absent in blood vessels, but detectable in the decidua. We found CB2 immunoreactivity in spot-like distribution in the amnion (Figure 3F-H).

#### FAAH immunoreactive staining patterns

FAAH immunoreactivity showed similar localization to CB2, as we found positive reaction in syncytiotrophoblasts and stromal fibroblasts (however, staining intensity was reduced compared to CB1 immunoreactivity). Immunoreactive FAAH was absent in blood vessels, but detectable in the decidua and less markedly in spot-like distribution in the amnion. FAAH immunoreactive staining intensity and localization was similar in normal and preeclamptic placenta (Figure 3I-K).

## Discussion

In this study, we determined CB1, CB2 and FAAH expressions in placental samples from healthy and preeclamptic women. According to our findings, CB1 expression measured by Western blotting was markedly stronger in preeclamptic placenta, and these findings were confirmed by strong CB1 immunoreactivity in various placental localizations. However, we did not find significant differences between preeclamptic and normal placenta in terms of CB2 and FAAH expressions and immunoreactivity.

In both normal and preeclamptic placenta, immunoreactive CB1, CB2 and FAAH were detected in syncytiotrophoblasts, the mesenchymal core and decidua. We found strong CB1 immunoreactivity in endothelial cells of blood vessels and moderate reaction in vascular smooth muscle cells, whereas CB2 and FAAH immunoreactivity was absent in these localizations. Regarding the chorionic plate, a less intensive CB1 immunoreactivity and spot-like positive CB2 and FAAH staining was observed in the amnion. Our findings were similar to the literature published earlier regarding ECS localization in human placenta [15-18].

Our results have demonstrated that CB1 expression and immunoreactivity was markedly higher in syncytiotrophoblasts and the mesenchymal core in preeclamptic samples compared to normal pregnancies. This is consistent with earlier reports, where strong immunopositivity for CB1 was found in trophoblast cells of placental samples of spontaneous miscarriage, although we did not detect lower FAAH signal in syncytiotrophoblasts as formerly reported [18]. These findings can be explained by the role of ECS in implantation and placental development. In rat pregnancy, major endocannabinoid levels increase gradually during pregnancy, while FAAH activity maintained constant during placentation [19]. As FAAH is responsible for protecting the fetus and placenta from high AEA levels [9], an early increase in AEA levels caused by higher NAPE-PLD activity can lead to impaired placental development due to defective trophoblast cell differentiation and invasion [20]. Trophoblast cells are responsible to invade the maternal decidua and forming the placenta. The tightly regulated invasion is affected by ECs, where both over- and underexpression of CB1 causes impaired trophoblast migration capabilities [11]. We hypothesize that high CB1 expression can result in poor placentation through insufficient trophoblast invasion, thereby leading to preeclampsia.

We also observed high CB1 expression and immunoreactivity in preeclamptic decidua. Earlier studies have shown that synthetic cannabinoids inhibit human decidual cell differentiation and mediate apoptosis through cAMP-dependent mechanism *via* CB1 activation [21]. Exposed cells expressed less decidualization-specific markers (such as prolactin, laminin and IGFBP-1) and showed significant cAMP level reduction, which led to morphological changes in decidual fibroblasts with DNA fragmentation. Therefore, elevated CB1 expression can result in increased apoptosis, thus impeding decidua establishment. However, it is also possible that the overexpression of CB1 exerts its effect mostly by disturbing decidua remodeling, thereby compromising placentation, leading to spontaneous abortion and preeclampsia.

Interestingly, according to the immunohistochemistry, preeclamptic placental samples also showed higher CB1 immunoreactivity in villous capillary endothelial and smooth muscle cells compared to normal pregnancy. This could be an adaptive response to poor placentation, as ECs can cause vasodilation through CB1, TRPV1 and NO-mediated or NO-independent mechanisms [22], thus enhancing the blood supply of the placenta. AEA exerts its vasorelaxant effect on endothelial cells in various ways, such as by upregulating the expression and activity of the inducible NO synthase (NO-mediated pathway) [23]. In the cerebral circulation, CB1 also has vasodilatory effects directly on vascular smooth muscle by inhibiting calcium entry through L-type calcium channels [24].

Abán et al. recently measured CB1 and FAAH expressions and localization in healthy and preeclamptic placenta [25]. They detected similar CB1 protein expression in both normal and preeclamptic tissues; however, NAPE-PLD expression was significantly enhanced, while FAAH was lower or undetectable in preeclampsia. These findings confirm the pathological activation of the ECS in preeclampsia, although the study does not report on decidual expression, as well as on expression and localization of CB2 protein. Differences between the latter and our results can be explained by the differences in gestational weeks of the preeclamptic group, in the severity of the disease or in antibodies used.

A limitation of our study is the lack of measurement of placental anandamide levels and NAPE-PLD expression. Furthermore, our study had a case–control design, thus direct causation cannot be determined. Nevertheless, abnormal placentation occurs in the first half of pregnancy, while the clinical syndrome only appears in the second, which rules out prospective studies on the placenta. It is also possible that increased CB1 expression is rather a consequence than a cause of preeclampsia, however there are no data in the literature demonstrating association between placental CB1 expression levels and PE risk factors or pathophysiological signals. Experimental studies are required to determine the causative role of increased placental expression of cannabinoid receptor 1 in preeclampsia.

## Conclusions

We observed markedly higher expression of CB1 protein in preeclamptic placental tissue. Increased CB1 expression might cause abnormal decidualization and impair trophoblast invasion, thus being involved in the pathogenesis of preeclampsia. Nevertheless, we did not find significant differences between preeclamptic and normal placental tissue regarding CB2 and FAAH expressions. While the detailed pathogenesis of preeclampsia is still unclear, the endocannabinoid system could play a role in the development of the disease.
